# Exascale applications: skin in the game

**DOI:** 10.1098/rsta.2019.0056

**Published:** 2020-01-20

**Authors:** Francis Alexander, Ann Almgren, John Bell, Amitava Bhattacharjee, Jacqueline Chen, Phil Colella, David Daniel, Jack DeSlippe, Lori Diachin, Erik Draeger, Anshu Dubey, Thom Dunning, Thomas Evans, Ian Foster, Marianne Francois, Tim Germann, Mark Gordon, Salman Habib, Mahantesh Halappanavar, Steven Hamilton, William Hart, Zhenyu (Henry) Huang, Aimee Hungerford, Daniel Kasen, Paul R. C. Kent, Tzanio Kolev, Douglas B. Kothe, Andreas Kronfeld, Ye Luo, Paul Mackenzie, David McCallen, Bronson Messer, Sue Mniszewski, Chris Oehmen, Amedeo Perazzo, Danny Perez, David Richards, William J. Rider, Rob Rieben, Kenneth Roche, Andrew Siegel, Michael Sprague, Carl Steefel, Rick Stevens, Madhava Syamlal, Mark Taylor, John Turner, Jean-Luc Vay, Artur F. Voter, Theresa L. Windus, Katherine Yelick

**Affiliations:** 1 Brookhaven National Laboratory, Upton, NY, USA; 2 Lawrence Berkeley National Laboratory, Berkeley, CA, USA; 3 Princeton Plasma Physics Laboratory, Princeton, NJ, USA; 4 Sandia National Laboratories, Albuquerque, NM, USA; 5 Los Alamos National Laboratory, Los Alamos, NM, USA; 6 Lawrence Livermore National Laboratory, Livermore, CA, USA; 7 Argonne National Laboratory, Argonne, IL, USA; 8 Pacific Northwest National Laboratory, Richland, WA, USA; 9 Oak Ridge National Laboratory, Oak Ridge, TN, USA; 10 Ames Laboratory, Ames, IA, USA; 11 Fermi National Accelerator Laboratory, Batavia, IL, USA; 12 SLAC National Accelerator Laboratory, Menlo Park, CA, USA; 13 National Renewable Energy Laboratory, Golden, CO, USA; 14 National Energy Technology Laboratory, Morgantown, WV, USA

**Keywords:** exascale, high-performance computing, computational science applications, numerical algorithms, machine learning, modelling and simulation

## Abstract

As noted in Wikipedia, *skin in the game* refers to having ‘incurred risk by being involved in achieving a goal’, where ‘*skin* is a synecdoche for the person involved, and *game* is the metaphor for actions on the field of play under discussion’. For exascale applications under development in the US Department of Energy Exascale Computing Project, nothing could be more apt, with the *skin* being exascale applications and the *game* being delivering comprehensive science-based computational applications that effectively exploit exascale high-performance computing technologies to provide breakthrough modelling and simulation and data science solutions. These solutions will yield high-confidence insights and answers to the most critical problems and challenges for the USA in scientific discovery, national security, energy assurance, economic competitiveness and advanced healthcare.

This article is part of a discussion meeting issue ‘Numerical algorithms for high-performance computational science’.

## 1. Introduction and background

The Exascale Computing Project (ECP), initiated in 2016 by the US Department of Energy (DOE), is an aggressive research, development and deployment (RD&D) project focused on the delivery of mission-critical applications, an integrated software stack and exascale hardware technology advances. These products are being deployed to DOE high-performance computing (HPC) facilities on pre-exascale and ultimately exascale computers, where they will address critical challenges for the USA in national security, energy assurance, economic competitiveness, healthcare and scientific discovery.

Exascale applications (and their companion co-designed computational motifs) are a foundational element of the ECP and are an important vehicle for delivery of consequential solutions and insight from exascale systems. The breadth of these applications runs the gamut: chemistry and materials; energy production and transmission; earth and space science; data analytics and optimization; and national security. Applications are built on underlying software technologies that play an essential supporting role in application efficacy on a broad range computing systems. An expanded and vertically integrated software stack is being developed to include advanced mathematical libraries and frameworks, extreme-scale programming environments and tools, and visualization libraries. ECP activities ensure a capable exascale computing ecosystem by integrating exascale applications, software technologies and hardware innovations into the DOE HPC facilities. The project also supports US HPC vendor R&D focused on innovative architectures for competitive exascale system designs. Further details on the overall scope and objectives of the ECP can be found at www.exascaleproject.org as well as in [1]; the focus herein is specifically on the exascale applications under development in the ECP.

Each ECP application is focused on targeted development to address a unique mission *challenge problem*, i.e. one that possesses a solution amenable to simulation insight, represents a strategic problem important to a DOE mission programme, and is currently intractable without the computational power of exascale computers. The ECP applications, and their associated exascale challenge problem, were carefully selected in 2016 based on five key criteria: technical review by external subject matter experts for confirmation that their challenge problem was significant and required exascale resources; alignment with DOE mission priorities via relevance of challenge problem to key DOE stakeholders; alignment with DOE national laboratory strategic priorities, i.e. mapping of application areas with laboratories' key capabilities across DOE; breadth of applications across computational motifs and engineering/science disciplines to maximize impact provided by ECP to both DOE and the broader community; and experience leveraging HPC systems, or the confidence that teams could hit ambitious exascale performance targets.

Tangible development progress requires close coordination among exascale application, algorithm and software development in order to address key application development challenges: porting to accelerator-based architectures; exposing parallelism at the level of millions of concurrent activities; coupling codes to create new multi-physics capabilities; adopting new mathematical approaches; algorithmic or model improvements; and leveraging optimized libraries. Summarized in the following are the exascale applications under development within the ECP and their challenge problem goals and impact.

## 2. Assessing application development success

Given the large investment by the DOE into ECP overall and specifically for ECP application development, rigorous project management processes for risk-informed, milestone-based planning, execution, tracking and assessment of the development activities are used throughout the project. The project overall also has a handful of metrics it must formally achieve for success, and these take the form of key performance parameters (KPPs) with specific quantified completion criteria. To assure progress is being made in accordance with ECP's target KPP metrics, the project undergoes deep-dive annual external reviews by its DOE stakeholders and subject matter experts.

Each ECP application has committed to achieving a specific KPP during the latter years of the project (2023–24), which falls into one of two groups (denoted as KPP-1 and KPP-2). KPP-1 quantitatively measures the increased capability of applications on exascale platforms compared with their capability on the HPC systems available at the start of the project (*ca* 2016). Each application targeting KPP-1 has defined a quantitative Figure of Merit (FOM) that represents the rate of ‘science work’ for their exascale challenge problem. FOM definitions are specific to an application area, and are reviewed both internally and externally (to ECP) to confirm that they are appropriate representations of capability improvements for that domain. Because exascale challenge problems cannot be executed on petascale resources, the FOM does account for differences in problem size, numerical precision, algorithm complexity and physical model enhancement to allow for an accurate measurement of the ultimate FOM improvement used to satisfy KPP-1. In ECP, KPP-1 applications must achieve greater than 50× performance improvement (as measured by their FOM) on their exascale challenge problem.

The ECP KPP-2 metric is intended to assess the successful creation of new exascale science and engineering DOE mission application capabilities. Applications targeting KPP-2 are required to define an exascale challenge problem that represents a significant capability advance in its area of interest to the DOE. These challenge problem targets are reviewed both internally and externally to confirm that they are impactful, challenging, tractable and of interest to a key DOE stakeholder. The distinguishing feature of KPP-2 applications relative to those targeting KPP-1 is the amount of new capability that must be developed to enable execution of the exascale challenge problem. An appropriate measure of success for these applications is whether the necessary capability to execute their exascale challenge problems is in place at the end of the project, not the relative performance improvement throughout the project.

ECP applications, then, do formally have ‘skin in the game’ via their commitment to achieving a specific and quantitative KPP-1 or KPP-2 metric, and this commitment is made several years in advance of when these metrics are actually measured (upon arrival of exascale systems).

## 3. Co-designed computational motifs

The goal of co-designed computational motifs in the ECP is to integrate a rapidly developing software stack with emerging hardware technologies, while developing software components that embody the most common patterns of application computation and communication, 13 of which are notable: dense linear algebra, sparse linear algebra, spectral methods, particles, structured grids, unstructured grids, Monte Carlo, combinational logic, graph traversal, graphical models, finite-state machines, dynamic programming, and backtrack and branch-and-bound [2]. The co-designed components will be integrated into the respective application software environments for testing, use and requirements feedback. This co-design process must balance application requirements with constraints imposed by the hardware and what is feasible in the software stack to facilitate performant exascale applications. The ECP incorporates six co-designed motif efforts (centres) as well as a ‘proxy applications’ effort whose mission is to develop proxy tools to explore algorithms, data structures/layouts, optimizations, etc., and the associated trade-offs on different architectures.

### (a) Proxy applications

Proxy applications (apps) are small, simplified codes that allow application developers to share important features of large applications without forcing collaborators to assimilate large and complex code bases. They are often used as models for performance-critical computations, but proxy apps can do more than just represent algorithms or computational characteristics. They also capture programming methods and styles that drive requirements for compilers and other elements of the tool chain. Within the ECP, application teams, co-design centres, software technology projects and HPC vendors all use proxy apps as a major mechanism to drive collaborations and co-design solutions for exascale challenges. To help accomplish this goal, an ECP proxy app suite composed of proxies that represent the most important features (especially performance) of exascale applications has been created. This suite is regularly updated and is released and distributed through the website: https://proxyapps.exascaleproject.org/.

An important component of the proxy applications effort is to help gauge the status and progress being made by the application software development teams towards computing their exascale challenge problems. The goal is to help keep the code developments on track, and to provide an outlet for expert guidance and feedback on programming issues such as performance portability over distinct machine designs, integration of methods that stand to impact code performance or scalability, and software quality. Computer experiments are coordinated with the developers to confirm production-scale reference problem execution on target DOE HPC platforms, and to experiment with weak and strong scaling behaviour of the codes on real inputs. Performance profiles of the execution efficiency for these experiments are created through the use of performance tools to help better understand code execution behaviour, to pinpoint inefficiencies and scaling issues, and to guide optimizations.

### (b) Online data analysis and reduction

A growing disparity between simulation speeds and I/O rates makes it increasingly infeasible for high-performance applications to save all results for offline analysis. By 2024, computers are expected to compute at 10^18^ ops s^−1^ but write to disk only at 10^12 ^bytes s^−1^: a compute-to-output ratio 200 times worse than on the first petascale systems. In this new world, applications must increasingly perform online data analysis and reduction—tasks that introduce algorithmic, implementation and programming model challenges that are unfamiliar to many scientists and that have major implications for the design of various elements of exascale systems. A co-design centre focused on online data analysis and reduction (*CODAR*) at the exascale addresses this issue [3]. Working closely with the ECP applications, CODAR targets both common data analysis and reduction methods (e.g. anomaly detection, feature tracking, compression) and methods specific to particular data types and domains (e.g. particle and structured finite-element methods). The team engages directly with providers of the ECP system software, programming models, data analysis and reduction algorithms, and applications in order to understand and guide trade-offs in the development of applications and software frameworks, given constraints relating to application development costs, application fidelity, performance portability, scalability and power efficiency. The goals of CODAR are to reduce the development risk for the ECP application teams by investigating crucial performance trade-offs related to the treatment of scientific results created by scientific models, produce high-performance implementations of data analysis and reduction methods, enable easy and efficient integration of those methods with applications, and contribute to the co-design of effective exascale applications and software.

### (c) Particles

The ECP Co-design Center for Particle Applications (*CoPA*) is addressing challenges for particle-based applications to run on upcoming exascale computing architectures. This scope is partitioned into four ‘sub-motifs’: short-range particle–particle interactions (e.g. those which often dominate molecular dynamics and smooth particle hydrodynamics methods), long-range particle–particle (e.g. electrostatic and gravitational) interactions, particle-in-cell (PIC) methods, and linear-scaling electronic structure and quantum molecular dynamics (QMD) algorithms. The crosscutting co-designed technologies fall into two types: proxy apps and libraries. Libraries are modular instantiations that multiple applications can use or be built upon. Cabana is a performance portable library for particle-based simulations (https://github.com/ECP-copa/Cabana). Applications include but are not limited to molecular dynamics (MD) with either short- and/or long-range interactions and various flavours of PIC methods, including applications to fluid and solid mechanics and plasma physics. Cabana provides particle data structures, algorithms and utilities to enable simulations on a variety of platforms including many-core architectures and GPUs. The PROGRESS library provides quantum chemistry solvers for QMD (https://github.com/lanl/qmd-progress). It uses the basic matrix library package (BML) which provides a common application programming interface for linear algebra and matrix functions on CPU-based and CPU–GPU architectures and is matrix-format independent (https://github.com/lanl/bml). Additionally, the SWFFT and fftMPI parallel fast Fourier transform libraries are available. Success is measured by the adoption by production codes (existing or newly developed), with both productivity and performance benefits.

### (d) Block-structured adaptive mesh refinement

A new framework, *AMReX*, supports the development of block-structured adaptive mesh refinement (AMR) algorithms for solving systems of partial differential equations, in simple or complex geometries, on exascale architectures [4]. Block-structured AMR provides the basis for the temporal and spatial discretization strategy for a large number of ECP applications in the areas of accelerator design, astrophysics, combustion, cosmology, multiphase flow, additive manufacturing (AM) and wind plant modelling. AMReX provides a unified infrastructure with the functionality needed for these and other AMR applications to be able to effectively and efficiently use exascale architectures. AMR reduces the computational cost and memory footprint compared to a uniform mesh while preserving accurate descriptions of different physical processes in complex multi-physics algorithms. AMReX supports algorithms that use particles and/or particle-mesh operations to represent component physical processes in addition to mesh-based solution approaches. Fundamental to block-structured AMR algorithms is a hierarchical representation of the solution at multiple levels of resolution. At each level of refinement, the solution is defined on the union of data containers at that resolution, each of which represents the solution over a logically rectangular subregion of the domain. Solution strategies vary from level-by-level approaches (with or without subcycling in time) with multilevel synchronization to full-hierarchy approaches, and any combination thereof. AMReX provides data containers and iterators that understand the underlying hierarchical parallelism for field variables on a mesh, particle data and embedded boundary (cut cell) representations of complex geometries. Both particles and embedded boundary representations introduce additional irregularity and complexity in the way data are stored and operated on, requiring special attention in the presence of the dynamically changing hierarchical mesh structure and AMR time-stepping approaches. AMReX also provides performance portability, enabling AMReX-based applications to move between CPU-based architectures and different hybrid CPU-accelerator systems with minimal changes to the application code itself.

### (e) Efficient finite-element discretization of PDEs on unstructured meshes

Efficient exploitation of exascale architectures requires a rethink of the numerical algorithms used in large-scale applications of strategic interest to the DOE. These architectures favour algorithms that expose ultra-fine-grain parallelism and maximize the ratio of floating-point operations to energy-intensive data movement. Many large-scale (and ECP) applications employ unstructured finite-element discretization methods, where practical efficiency is measured by the accuracy achieved per unit computational time. One of the few viable approaches to achieve high performance, in this case, is to use matrix-free high-order finite-element methods, since these methods can both increase the accuracy and/or lower the computational time due to reduced data motion.

To achieve this efficiency, high-order methods use mesh elements that are mapped from canonical reference elements (hexes, wedges, pyramids and tetrahedra) and exploit, where possible, the tensor-product structure of the canonical mesh elements and finite-element spaces. Through matrix-free partial assembly, the use of canonical reference elements enables substantial cache efficiency and minimizes extraneous data movement in comparison to traditional low-order approaches [5].

The co-design Center for Efficient Exascale Discretizations (*CEED*) is developing the next-generation discretization software and algorithms that will enable a wide range of finite-element applications to run efficiently on future hardware (https://ceed.exascaleproject.org/). High-order methods are the logical choice for this, from a mathematical (higher-quality simulations) perspective, as well as from HPC (better performance) and risk mitigation perspectives (range of orders provides flexibility in the uncertain exascale hardware and software environments). Their efficiency extends to problems with unstructured non-conforming mesh refinement and general curved meshes and includes low-order finite-element discretizations as a special case. The CEED work covers all of these topics, including the full low- to high-order spectrum of discretizations, allowing software to be easily integrated with low-order applications while enabling such applications to naturally transition from low- to high-order methods.

### (f) Combinatorial methods

Combinatorial algorithms in general and graph algorithms in particular play a critical enabling role in numerous scientific applications [6]. The irregular memory access nature of these algorithms makes them one of the hardest algorithmic kernels to implement on parallel systems [7]. The co-design Center for Graph and Combinatorial Methods for Enabling Exascale Applications (*ExaGraph*) is developing methods and techniques for efficient implementation of key combinatorial algorithms carefully selected from a set of exascale applications. There are three dimensions to this effort: (i) exascale applications that drive the selection of combinatorial kernels and integration of software tools developed, such as computational biology, computational chemistry and climate science; (ii) combinatorial (graph) kernels that play a crucial enabling role in the chosen application areas, such as graph traversals, graph matching, graph colouring, graph clustering and graph partitioning; and (iii) efficient implementations on hierarchical distributed-memory architectures representative of exascale platforms. Previous and ongoing efforts from ExaGraph have resulted in the design and implementation of several variants of graph matching with important applications in sparse linear algebra; graph clustering with key enabling role in computational biology; graph colouring with applications in algorithmic differentiation and algebraic multigrid methods; sparse matrix ordering methods with applications in computational chemistry; and graph partitioning with numerous applications in a variety of scientific computing contexts. The current efforts of ExaGraph are focused on porting and optimization of graph algorithms for accelerator-based architectures that not only pose significant challenges but also have ample scope for improvements in runtime and middleware systems in support of efficient execution of graph algorithms on the exascale systems. Parallel sparse matrix kernels and ordering techniques for the efficient solution of large, sparse, non-symmetric systems of linear equations is also a target of opportunity for ExaGraph.

### (g) Machine learning

The ECP must leverage the revolution in what is variously termed machine learning, statistical learning, computational learning and artificial intelligence (henceforth referred to as machine learning or ML). New ML technologies can have profound implications for computational and experimental science and engineering and thus for the exascale computing systems being developed to support those disciplines. Not only do these learning technologies open up exciting opportunities for scientific discovery on exascale systems, they also appear poised to have important implications for the design and use of the same exascale computers themselves. The ECP co-design centre devoted to ML (*ExaLearn*) provides exascale ML software for use by the ECP applications, other ECP co-design centres, and DOE experimental facilities and leadership class computing facilities. Working closely with ECP applications, ExaLearn is focused on a co-design process that targets learning methods common across these applications. These include deep neural networks of various types (e.g. recurrent neural networks, convolutional neural networks, generative adversarial networks), kernel and tensor methods, decision trees, ensemble methods, graphical models and reinforcement learning methods. ExaLearn is identifying the fundamental ML challenges associated with ECP and concentrating efforts on the development of scalable ML technologies for the analysis of data generated by exascale applications and DOE user facilities as well as to guide the optimal selection and steering of (i) complex computer simulations (e.g. current exascale application projects) and (ii) experiments (e.g. at DOE facilities including light sources, the National Ignition Facility and accelerators). Key to success in this endeavour is a deliberate focus on verification and validation and uncertainty quantification with a solid determination of generalization errors. A unifying principle is that of using exascale ML to improve the efficiency and effectiveness both of DOE computing resources and experimental facilities.

## 4. Chemistry and materials applications

The chemistry and materials applications area focuses on simulation capabilities that aim to precisely describe the underlying properties of matter needed to optimize and control the design of new materials and energy technologies. The underlying physics that governs these application areas is computationally challenging, e.g. capturing quantum effects can introduce significant communication non-locality and computational complexity. Efficiently scaling these methods to exascale is especially challenging.

### (a) Nuclear physics: lattice gauge quantum chromodynamics

The strong interactions between quarks and gluons generate 99% of the mass in the visible universe. Understanding these interactions and the phenomena that underly this observation is the central goal of nuclear physics [8,9]. The *LatticeQCD* application is implementing scalable quantum chromodynamics (QCD) algorithms to realistically simulate the atomic nucleus in order to reveal a deeper understanding about the fundamental organization of matter at the subatomic level.

Atomic nuclei and most particles are tightly bound composites of quarks and gluons. The fundamental interaction of these quarks and gluons is known as the nuclear or strong force, which is one of the four fundamental forces of nature (i.e. strong, weak, electromagnetic and gravity). The modern theory of these nuclear interactions is QCD, and HPC is required to predict the consequences of this underlying theory. The properties of the resulting bound states and the nature of their strong, highly nonlinear interactions is the central focus of nuclear physics and the context in which high-energy physics research must be conducted.

The couplings between the quarks and the W, Z and Higgs bosons lie at the heart of the Standard Model of particle physics and can be studied, often with exquisite precision, by measuring the properties of the bound states formed from these quarks and gluons. QCD is the fundamental theory of the interactions between quarks and gluons and can be solved only through massive computation. Over the past three decades, QCD computations have been a driver of, and benefited from, the spectacular advances in HPC [10]. Computing at the exascale is essential to reach two decadal challenges of central importance to nuclear and high-energy physics.

The advance to exascale capability over the coming decade offers exciting opportunities for ground-breaking discoveries in high-energy and nuclear physics. Exascale computing has the potential to realistically simulate the atomic nucleus and to discover the first harbingers of new laws of nature [11,12,13], revealing a deeper theory which underlies the present ‘elementary’ particles [14]. These possibilities can be achieved if new and impending advances in computer science via the ECP can be harnessed to provide a software framework that allows lattice QCD applications to efficiently exploit exascale architectures and application scientists to refine that application as new challenges and ideas emerge.

### (b) Chemistry: tackling chemical, materials and biomolecular challenges

The design of feedstock for the efficient production of biomass and the design of new catalysts for the efficient conversion of biomass-derived intermediates into biofuels are two science challenges that are critical to the development of advanced biofuels. A major goal of DOE's advanced biofuels programme is to develop fuels that can use the existing infrastructure and replace existing fuels on a gallon-for-gallon basis. However, producing high-quality biofuels in a sustainable and economically competitive way is technically challenging, especially in a changing global climate. The *NWChemEx* application [15] will provide the high-fidelity modelling capabilities needed to assist in the development of new biomass feed stocks as well as the efficient catalytic conversion of the resulting biomass-derived intermediates into biofuels and other bioproducts. In addition to providing the means to solve the biofuel challenge problems, NWChemEx will enable exascale computers to be applied towards the development of new materials for solar energy conversion and next-generation batteries, simulation of the chemical processes in combustion, prediction of the transport and sequestration of energy byproducts in the environment, development of a science of synthesis and design of new functional materials.

NWChemEx aims to provide a framework for a community-wide effort to develop next-generation molecular modelling capabilities that support a broad range of chemistry research on computing systems ranging from terascale workstations and petascale servers to exascale systems. NWChemEx is based on NWChem [16], an open-source computational chemistry program that is being actively developed by an international consortium of scientists. NWChem is a high-performance parallel code that provides a broad range of capabilities for modelling molecular systems. The NWChemEx project is re-designing and re-implementing NWChem for pre-exascale and exascale computers.

NWChemEx is developing high-performance, scalable implementations of three major physical models: Hartree–Fock method; Density Functional Theory (DFT) methods and a robust suite of canonical, domain localized and explicitly correlated coupled-cluster (CC) methods. The latter CC methods are essential for achieving chemical accuracy for the molecular processes of interest in the development of biofuels. In addition, the NWChemEx application is developing Density Functional Embedding Theory to describe the larger environment surrounding an active site. Embedding techniques provide a natural and mathematically sound basis for seamlessly integrating subsystems with different electronic structure representations, enabling the active site of interest to be described with high-accuracy CC methods, while using a lower fidelity method such as DFT to describe the impact of the environment on the molecular processes in the active site.

### (c) Chemistry: heterogeneous catalysis and new catalyst design

Heterogeneous catalysis and the design of new catalysts is a grand challenge problem in computational chemistry that requires the availability of exascale computers. The *GAMESS* application [17] is extending methods and algorithms based on chemical fragmentation methods and coupling these with high-level electronic structure methods including coupled-cluster theory and quantum Monte Carlo in order to solve this problem. Defining a representative heterogeneous catalysis problem comprising mesoporous silica nanoparticles (MSNs), GAMESS will demonstrate the capability to model physical systems requiring chemical interactions involving thousands of atoms, indicating a new ability to model complex chemical processes.

To take full advantage of exascale architectures, the GAMESS electronic structure software must be capable of exploiting multiple layers of parallelism and take advantage of emerging architectures that dramatically lower the energy/power cost without significant deterioration of time to solution. This work is developing *ab initio* methods, based on fragmentation methods that have been shown to scale beyond the petascale, combined with quantum Monte Carlo. To achieve exascale performance, GAMESS is being refactored to take advantage of modern computer hardware and software, and the capabilities of the C++ libcchem code is being greatly expanded. Concurrently, performance analysis is underway for the broad array of electronic structure methods in GAMESS on current and emerging architectures to assess their ability to decrease time to solution while decreasing energy demands.

GAMESS will be brought to bear on the heterogeneous catalysis problem, specifically using MSNs. MSNs are highly effective and selective heterogeneous catalysts for a wide variety of important reactions. MSN selectivity is provided by ‘gatekeeper’ groups that allow only desired reactants to enter the pore and keeping undesirable species out. The presence of a solvent further complicates the problem. Accurate electronic structure calculations are needed to deduce the reaction mechanism(s), including the effects of various solvents and to subsequently design even more effective catalysts. While it is common to approximate a system like this with a small model, a recent computational study of MSN catalysis of carbinolamine formation demonstrated that small proxy models are inadequate both qualitatively and quantitatively. The GAMESS application is targeting simulation of both the energetics and dynamics on a model reaction with an adequate representation of the MSN pore, requiring thousands of atoms with an appropriate basis set (e.g. 5 K heavy atoms requires more than 500 K basis functions, not including the hydrogen atoms, and the reacting and solvent molecules). The energy surface will be mapped via GAMESS calculations using the effective fragment molecular orbital method [18] plus resolution of identity second-order Mollet–Plesset perturbation methodology, with refined calculations adding a CC or quantum Monte Carlo (QMC) approach for accurate reaction rates. Pore selectivity dynamics will be computed with a molecular dynamics (MD) approach requiring approximately 10 K energetics type calculations using a fragment molecular orbital methodology.

### (d) Molecular dynamics for materials in the extremes

Molecular dynamics, the numerical integration of the equations of motion of atoms, is a cornerstone of computational science. However, MD is frequently prevented from achieving complete scientific success by the inability to simultaneously reach the necessary length and time scales while maintaining sufficient accuracy. While the raw computing power available at the exascale should allow for a dramatic extension of the range of applicability of MD, conventional massively parallel codes suffer from poor strong scalability. This implies that a simple scale-up of current practices would only enable the simulation of much larger systems (billions or trillions of atoms) but would do little to improve current time scales (ns) and accuracy (empirical potentials). As most challenging problems instead require accessing different regions in the accuracy (A), length (L) and time (T) simulation space (ALT), one of the community's key tools, MD, is in danger of missing out on the exascale revolution.

The *EXAALT* application (http://gitlab.com/exaalt/) combines three state-of-the-art codes—LAMMPS, LATTE and ParSplice—into a unified tool that will leverage exascale platforms efficiently across all three dimensions of the ALT space. The new integrated capability is composed of three software layers. First, a task management layer enables the creation of large numbers of MD tasks, their management through task queues, and the storage of results in distributed databases. This layer is used to implement various replica-based accelerated MD techniques that enable a significant extension of the simulation time scales though a time-wise parallelization approach [19], as well as other complex MD workflows. The second layer is a powerful MD engine based on the LAMMPS code [20]. It offers a uniform interface through which the different physical models can be accessed. The third layer provides a wide range of physical models. In addition to a large number of empirical potentials implemented in LAMMPS, EXAALT provides high-performance implementations of electronic structure-driven MD at the Density Functional Tight Binding level through the LATTE code (https://github.com/lanl/LATTE), as well as to high-accuracy machine-learned potentials such as spectral neighbour analysis potentials (SNAP) [21].

EXAALT is tackling two materials-in-extremes challenge problems, one targeting nuclear fuel materials and the other targeting first wall materials in future fusion reactors. A primary concern for nuclear fuel is material integrity: as the fuel burns, radiation damage and fission gases accumulate, causing swelling of the fuel, pellet–clad interactions and increased pressure on the clad. Because current burn-up levels are predicated on understanding how the fuel evolves, improved models of fission gas evolution offer the potential for extracting more energy from the fuels. Solving this challenge required a significant advance in carrying out high-accuracy, electronic structure-driven MD simulations on the time scales necessary to observe diffusion of defects while accounting for their changing charge-state. The solution to this problem requires EXAALT development at exascale to increase the currently accessible time scales by 1000×. The second challenge problem, supporting the realization of fusion as a commercially attractive twenty-first century energy source, targets the design of advanced structural materials capable of sustained operation in an extreme environment with high temperatures and high fluxes of helium, hydrogen isotopes and neutrons. The performance demands on plasma-facing components of future fusion power plants are beyond the capability of current materials. Tungsten is a leading divertor material candidate. Experiments on this material, however, indicate the possibility of substantial surface modification in tungsten exposed to low-energy plasma containing helium. These modifications to the microstructure can lead to premature failure of the materials or quench the fusion reaction by cooling and de-stabilizing the plasma. Solving this problem requires a dramatic extension of the reach of large-size long-time MD simulations that EXAALT deployed on exascale hardware will provide.

### (e) Transforming metal additive manufacturing

Additive manufacturing (AM) is revolutionizing manufacturing, allowing construction of complex parts not readily fabricated by traditional techniques [22]. In addition, AM offers the possibility of constructing ‘designer materials’ by adjusting process control variables to achieve spatially varying physical properties [23]. AM is a unique application area due to its strategic importance to both US industry and federal agencies. Although there has been significant interest and investment in AM, the fraction of this investment devoted to modelling and simulation is relatively small and not focused on the development of high-fidelity predictive models but instead on reduced-order models for industry use. The *ExaAM* application represents a unique opportunity to use exascale simulation to enable design of AM components with location-specific properties and acceleration of performance certification.

In AM, a geometric description of the part is processed into two-dimensional slices. A feedstock material is melted and the part is built layer-by-layer. In metal AM, the feedstock is often in wire or powder form, and the energy source is a laser or electron beam. ExaAM is focusing on powder bed processes, where each layer is approximately 20–50 µm thick. The physical processes involved in AM (heat transfer, fluid flow, melting and solidification, microstructure evolution and solid–solid phase transformation) are similar to those of welding—a field with a wealth of experimental, modelling, simulation and characterization research over the past decades. Unfortunately, the simulation tools developed for welding and other similar processes, while calibrated and approaching predictive capability, are inadequate for AM processes due to higher solidification rates and increased significance of non-equilibrium effects [24].

A key goal of the ExaAM application is to remove those limitations by coupling high-fidelity mesoscale simulations within continuum process simulations to determine microstructure and properties using local conditions. Typically, thermo-mechanical finite-element models are employed at the macroscopic part scale [25]; finite volume or finite-element models for fluid dynamics and heat transfer to capture the melt pool dynamics and solidification at millimetre scales [26]; mesoscale approaches to simulate melting, solidification and microstructure formation and solid–solid phase transformation at the micrometre scale [27–29]; and polycrystal plasticity models to develop the microscale mechanical property relationships [30]. ExaAM will develop and deploy a collection of multi-scale, multi-physics simulation capabilities for performing process-aware performance modelling of AM parts using locally accurate properties predicted from microstructures that develop based on local processing conditions.

### (f) Predictive quantum-mechanics simulation of strongly correlated materials

Materials design has progressed from the study of simple bulk properties to targeting collective effects in strongly correlated materials such as magnetic ordering, phase transitions and quantum coherence. This requires a fundamentally different set of computational tools that have been used in the past, and the open-source *QMCPACK* application [31] is employing QMC methods to attack this problem since they robustly deliver highly accurate calculations of complex materials that do not artificially bias solutions of a given character. With exascale computing, QMC has the potential to attain a significant increase in the number of modelled atoms and electrons in metal oxide systems that will fill missing gaps in experimental data and lead to advances in materials and nanoscience.

The ability to computationally design, optimize or understand the properties of energy-relevant materials is fundamentally contingent on the existence of methods to accurately, efficiently and reliably simulate them. Quantum-mechanics-based approaches must necessarily serve as a foundational role, since only these approaches can describe matter in a truly first-principles (parameter-free) and robust manner. The few approximations in QMC methods, with increased computer power, can be tested and systematically reduced, which is not possible with other first-principles methods.

The trade-off is that the computational demands of QMC are large. As an example, the use of petascale computers has allowed calculations of the magnetic exchange in copper oxide high-temperature superconductors, but the calculations are restricted to simplified models with only tens of atoms. A realistic model of actual superconductors would require at least several hundreds of atoms to include the crucial dopant atoms. The QMCPACK exascale challenge problem is to simulate transition metal oxide systems of approximately 1000 atoms to 10 meV statistical accuracy, with 50 times speed-up in time to solution over current petascale machines. Achieving this goal requires efficiently mapping the algorithms to exascale architectures and efficiently using on-node memory. Besides improving the main application, the project has created a proxy application and demonstrated viability of achieving the necessary speed-ups on accelerated platforms using both Kokkos and OpenMP target-based implementations.

The additional power and parallelism of exascale QMC will provide the essential predictive and quantitative capability for these and related materials that lie well beyond the capabilities of existing methods. Exascale provides the opportunity for highly impactful and enabling benchmark accuracy calculations on these materials, providing the reference calibration data that is missing from essentially all quantum-mechanics-based materials calculations today. This capability will be highly useful across the materials sciences, nanoscience and physics communities, particularly where experimental data are costly or difficult to obtain.

## 5. Energy applications

The energy applications area focuses on M&S of existing and future technologies for the efficient and responsible production of energy to meet the growing needs of the USA. Applications in this area generally require detailed modelling of complex facilities and multiple coupled physical processes. Their goal is to help overcome obstacles to the efficient and safe delivery of energy.

### (a) Predictive wind plant flow physics

A key to achieving wide-scale deployment of wind energy is understanding, predicting and reducing plant-level energy losses from a variety of physical flow phenomena. High-fidelity modelling, coupled with HPC, offers the most expedient path to drive significant reductions in the cost of wind energy through dramatic improvement in the understanding of fundamental flow physics governing whole wind plant performance, including wake formation, complex-terrain impacts and turbine–turbine interactions through wakes [32]. Thus, the exascale challenge [33] is the predictive simulation of a wind plant composed of many multi-MW wind turbines sited within a 10 × 10 km^2^ area with complex terrain, involving simulations with O(100) billion grid points. These predictive, physics-based, high-fidelity computational models, validated with targeted experiments, will drive innovation in the blade, turbine and wind plant design processes by providing a validated ‘ground truth’ foundation for new turbine design models, wind plant siting, operational controls and reliably integrating wind energy into the grid.

The *ExaWind* application (https://www.exawind.org/) goal is to advance fundamental understanding of the flow physics governing whole wind plant performance. Greater use of abundant wind resources for electric power generation, reaching 30% of US electrical supply, will have profound societal and economic impact: strengthening US energy security through greater diversity in its energy supply, providing cost-competitive electricity to key regions across the country, reducing greenhouse gas emission and reducing water used in thermo-electric power generation.

ExaWind embodies a systematic development of modelling capability and computational performance and scalability, building progressively from predictive petascale simulations of a single turbine (where the detailed blade geometry is resolved, meshes rotate and deform with blade motions, and atmospheric turbulence is realistically modelled) to a multi-turbine array in complex terrain. The new ExaWind capability will establish a virtual wind plant test bed that will revolutionize the design and control of wind farms and result in a significant advance in the ability to predict the response of wind farms to a wide range of atmospheric conditions.

The ExaWind challenge problem is a predictive simulation of a wind farm with tens of megawatt-scale wind turbines dispersed over an area of 50 km^2^. The goal is to capture crucial phenomena that are under-resolved in today's models, including wake formation, complex-terrain impacts, wake–atmosphere interaction, turbine–turbine interaction and blade boundary-layer dynamics. This target requires an M&S capability that resolves turbine geometry and uses adequate grid resolution (down to micrometre scales within the blade boundary layers). The resolution must capture the upstream chord-scale atmospheric turbulent eddies, generation of near-blade vorticity and propagation and breakdown of this vorticity, within the turbine wake to a distance of many rotor diameters downstream. This application uses the Nalu-Wind computational fluid dynamics (CFD) code and the OpenFAST turbine-simulation code that have been specifically designed for wind turbine and wind farm simulations. The simulation will require a hybrid Reynolds-averaged-Navier–Stokes—large-eddy-simulation (LES) turbulence model, fluid–structure interaction and atmospheric turbulent flow.

### (b) Transforming combustion science and technology

Aggressive US goals for significantly reducing petroleum use and greenhouse gas emissions require major improvements in all aspects of our nation's energy use. Combustion processes have historically dominated electrical power production and transportation systems. Despite major advances in improving the efficiency and reducing the costs of alternative energy sources, combustion-based systems are projected to dominate the marketplace for decades. Consequently, these systems need to be optimized for energy efficiency and reduced emissions.

The motivating problem that anchors the *Pele* application is a sufficiently realistic simulation of the in-cylinder processes in an internal combustion engine using low-temperature combustion, for which reactivity-controlled compression ignition (RCCI) is the exemplar. This exascale-class simulation will address key scientific questions regarding mixture formation effects, multi-stage ignition of a diesel surrogate fuel, lifted flame stabilization, jet re-entrainment affected by cylinder-wall geometry and emissions. The simulation will account for isentropic compression, subsequent injection of the high-reactivity fuel, and combustion processes in a compression ignition engine. Necessary physics include gas compression and models of fuel injection process, spray vaporization (injection of liquid fuel sprays into high-pressure conditions), mixing and combustion processes: autoignition, flame propagation, soot and thermal radiation, all in a non-trivial engine geometry. The scenario involves kinetically controlled processes in turbulent combustion including ignition, extinction and emissions.

To address these physical phenomena, Pele implements a hybrid LES plus Direction Numerical Simulation (DNS) approach in both the compressible and low-Mach limits where, using the machinery of AMR, length-scale refinement to the DNS limit will be invoked where necessary to capture turbulence/chemistry interactions while restricting resolution to that required for a high fidelity LES model far from the flame. The Pele application is therefore structured around a novel hybrid combination of first-principles DNS and near first-principles (DNS/LES) M&S capabilities to advance understanding of fundamental turbulence–chemistry interactions in device-relevant conditions. Many of the turbulence–chemistry interactions, e.g. ignition processes, are sensitive to the nuances of low-temperature chemistry, and hence, an automated framework is also under development to generate ‘drop-in’ chemical mechanisms in Pele with unprecedented accuracy. The exascale challenge problem, performing high-fidelity simulations of the relevant processes in a low-temperature and thermodynamically favourable RCCI internal combustion engine, holds the promise of unlocking ground-breaking engine efficiencies (relative to existing engines) while maintaining engine operation in a regime that limits pollutant formation.

### (c) Nuclear energy: small modular reactors

Small modular reactors (SMRs) and advanced reactor concepts will deliver clean, flexible, reliable and affordable electricity while avoiding the traditional limitations of large nuclear reactor designs, such as high capital costs and long construction timelines. Current advanced reactor design approaches leverage decades of experimental and operational experience of the US nuclear fleet and are informed by calibrated numerical models of reactor phenomena. The *ExaSMR* application generates virtual reactor design simulation datasets with high fidelity, coupled-physics models for reactor phenomena that are truly predictive, reflecting as much ‘ground truth’ as experimental and operational reactor data. ExaSMR virtual designs can accelerate the currently cumbersome advanced reactor concept-to-design-to-build cycle that has constrained the nuclear energy industry for decades. ExaSMR can also provide an avenue for validating existing industry design and regulatory tools.

ExaSMR integrates the most reliable and high confidence numerical methods for modelling operational reactors, namely the reactor's neutron state with Monte Carlo neutronics and the reactor's thermal fluid heat transfer efficiency with high-resolution CFD, and all for efficient execution on exascale systems. ExaSMR builds on a base of simulation applications that have demonstrated high efficiency on current petascale-class leadership computing systems.

ExaSMR's exascale challenge problem will open the door to high confidence prediction of advanced reactor conditions, such as during power ascension via the initiation of natural circulation of the coolant flow through a small reactor core and its primary heat exchanger. The exascale software orchestrating this simulation, known as ENRICO, ensures intimate coupling of CFD [34] and Monte Carlo neutron transport modules through a common interface that supports multiple exascale simulation technologies: one [35] targeting the exascale Frontier system at Oak Ridge National Laboratory and the other [36] targeting the exascale Aurora system at Argonne National Laboratory.

Exascale neutron transport simulations enabled by ExaSMR will accommodate a full-core SMR model, which typically has approximately 40 fuel assemblies (each with approx. 300 fuel rods). The Monte Carlo portion of the simulation will orchestrate unprecedented accuracy via 10B particles per eigenvalue iteration with pin-resolved reaction rates having three radial tally regions and 20 axial levels, and approximately 150 nuclides and eight reactions per nuclide in each tally region. Exascale CFD requirements for ExaSMR will include assembly bundle mesh models with momentum sources from a resolved representative spacer grid and full-core mesh having at least 40M elements and 22B degrees of freedom. With these ExaSMR capabilities in place, exascale reactor modelling capabilities will help inform and accelerate, at a high confidence level, the design of advanced and small modular reactors with unprecedented resolution and improve the modelling fidelity of the relevant complex physical phenomena occurring.

### (d) Performance prediction of multiphase energy conversion devices

Carbon capture and storage (CCS) technologies such as oxy-fuel combustion, chemical looping combustion and post-combustion capture offer the most promising approaches for reducing CO_2_ emissions from fossil fuel power plants. Large-scale commercial deployment of novel CO_2_ capture technologies requires understanding of how to scale laboratory designs of multiphase flow reactors to industrial sizes. However, the direct scale-up of such reactors is known to be unreliable and the current approach requires building and testing physical systems at increasingly larger, intermediate scales. The cost in both dollars and development time to build and extensively test systems at multiple intermediate scales is prohibitive. High-fidelity computational tools that use exascale computing power to model emerging CCS technologies help to enable the design and optimization of these systems, which is critical to control costs and reduce the risk of designs failing to meet performance standards.

The *MFIX-Exa* application (visit https://mfix.netl.doe.gov/ for information on MFIX) specifically targets scale-up of chemical looping reactors (CLRs). The 50 kW CLR at the National Energy Technology Laboratory (NETL) is being used for validating a CLR model based on *MFIX-Exa*. Chemical looping combustion occurs in two reactors that avoid the direct contacting of fuel and air. A fuel reactor uses oxygen from solid oxygen carriers, such as metal oxides, instead of air to combust fossil fuels, such as methane. An air reactor then regenerates the spent oxygen carrier with oxygen from air. The air reactor produces a hot air stream that is used to raise steam to drive a turbine for power generation; the fuel reactor produces gases from which CO_2_ can be easily captured.

*MFIX-Exa* employs a new scalable CFD Discrete Element Model (CFD-DEM) approach, representing the next generation of a highly successful and widely used NETL-based MFIX application suite. CFD-DEM is an approach that allows for tracking of individual particles (DEM portion) within a continuum fluid phase (CFD portion). Until ECP, the focus of existing *MFIX* CFD-DEM efforts has been on development and validation of physical models within a relatively traditional (legacy) computational framework. *MFIX-Exa* project, which integrates expertise in HPC and modern algorithms directly with modern multiphase flow models, is on track in the ECP to substantially outperform the legacy *MFIX* approach.

The exascale challenge problem is a CFD-DEM simulation of NETL's 50 kW CLR with 5 billion DEM particles for a sufficiently long physical time, i.e. until exit gas compositions reach a pseudo-stationary state, enabling the evaluation of reactor performance. The challenge problem requires representing the full-loop CLR geometry, covering various gas–solids flow regimes occurring in the CLR (bubbling bed, riser, cyclone, standpipe and L-valve), including interphase momentum, mass and energy transfer. Without the capabilities of *MFIX-Exa* at exascale, it is not possible to resolve the distribution in particle-scale properties (size, density, chemical conversion) in simulations of pilot-scale gas–solids reactors. This capability will enable a number of other applications such as the design and optimization of gas–solids reactors required for process intensification and modularization. The 1000× increase in the number of particles over the current state of the art in CFD-DEM will unlock the ability to simulate a host of new industrially relevant problems, e.g. agriculture, chemical, energy, mining, petrochemical and pharmaceutical.

### (e) Whole device modelling of magnetically confined fusion plasmas

The Whole Device Model application (*WDMApp*) strives to develop a high-fidelity model of magnetically confined fusion plasmas, which is urgently needed to plan experiments on ITER and optimize the design of future next-step fusion facilities. These devices will operate in high-fusion-gain physics regimes not achieved by any of the current or past experiments, making advanced and predictive numerical simulation the most viable tool for the task. WDMApp is focused on building the main driver and coupling framework for the more complete Whole Device Model (WDM), the ultimate goal being a comprehensive computational suite that incorporates all relevant physical phenomena required to model a magnetically confined fusion reactor with high confidence, an important goal for predictive fusion M&S as stated in [37]. The main driver for the WDM is the coupling of two advanced and highly scalable gyrokinetic codes, XGC and GENE. The former is based on a PIC formulation optimized for treating the edge plasma, while the latter is based on a continuum formulation optimized for the core plasma. WDMApp will take advantage of the complementary nature of these two applications to build the most advanced and efficient whole device kinetic transport kernel for the WDM.

A major thrust of the WDMApp is the coupling framework EFFIS 2.0 (End-to-end Framework for Fusion Integrated Simulation 2.0), which is being developed for exascale and optimized for coupling the physics modules to be incorporated in the WDM. The current MPI+X implementation with the ‘first-mover’ GENE and XGC applications is being enhanced with communication-avoiding methods, task-based parallelism, *in situ* analysis with resources for load optimization workflows and deep memory hierarchy-aware algorithms.

The WDMApp exascale challenge problem is the high-fidelity simulation of whole device burning plasmas applicable to a high-confinement (H-mode) advanced tokamak regime, specifically, an ITER steady-state plasma which aims to attain 10-fold energy gain. The physics objective is to predict one of the most important indicators for energy confinement in the H-mode, namely the plasma pressure ‘pedestal’ height and shape. Realization of the H-mode, with high edge plasma pressure and mild pedestal gradient, is critical to the performance and success of ITER. Efficiency of the fusion burn is virtually determined by the height of the pressure pedestal at edge. The strategy is to use WDMApp and its ability to couple the continuum code GENE in the core region and the PIC code XGC at the edge.

The resulting exascale application will be unique in its computational capabilities and have potentially transformational impact in fusion science, e.g. studying a much larger and more realistic range of dimensionless plasma parameters than ever before and the rich spectrum of kinetic micro-instabilities that control the quality of energy confinement in a toroidal plasma (including tokamaks as well as stellarators), with the core and the edge plasma strongly coupled at a fundamental kinetic level based on the gyrokinetic equations. Results on code-coupling algorithms in the electrostatic approximation (using a Boltzmann response for electrons) are encouraging [38].

### (f) Advanced particle accelerator design

Particle accelerators are used in many areas of fundamental research, as evidenced by a total of 30% of all Nobel prizes in physics since 1939, and four of the last 14 Nobel prizes in chemistry, having been enabled by this technology. Among the candidate new technologies for compact accelerators, the advent of plasma-based particle accelerators stands apart as a prime game-changing technology. The development of these devices depends critically on high-performance, high-fidelity modelling to capture the full complexity of acceleration processes that develop over a large range of space and time scales. The *WarpX* application (https://github.com/ECP-WarpX/WarpX) is developing a plasma accelerator model that enables the exploration of outstanding questions in the physics of the transport and acceleration of particle beams in long chains of plasma channels. These new breeds of virtual experiments, not possible with present technologies, will bring huge savings in the research leading to the design of a plasma-based collider, and even bigger savings via reliable characterization of the accelerator before it is built.

For most applications, the size and cost of the accelerators are limiting factors that can significantly impact the funding of projects or adoption of solutions. The requisite accelerator physics simulations are extremely computationally intensive, due to the need to resolve the evolution of a driver (laser or particle beam) and an accelerated beam into a structure that is orders of magnitude longer and wider than the accelerated beam. Studies of various effects, including injection, emittance transport, beam loading, tailoring of the plasma channel and tolerance to non-ideal effects (jitter, asymmetries, etc.), crucial for the design of high-energy colliders, will necessitate a series of tens to hundreds of runs. This requirement imposes orders-of-magnitude simulation speed-up over the present state of the art, which is possible only by combining the power of exascale computing with the most advanced computational techniques.

This application combines the PIC code Warp technology [39] and the AMR framework AMReX into a new code (WarpX), all the while porting and optimizing the software to exascale platforms. WarpX incorporates the most advanced algorithms that have been developed and validated by the lead teams, including, among others, the optimal Lorentz boosted frame approach [40], scalable spectral electromagnetic solvers [41] and mitigation methods for the numerical Cherenkov instability [42]. To ensure speed and scalability, WarpX takes advantage of the latest features developed in portable vectorization algorithms and hierarchical parallelism, as well as AMReX's dynamic gridding capabilities, to load balance the combined computational work associated with both the particles and the mesh. The new software will enable the exploration of outstanding questions in the physics of the transport and acceleration of particle beams in long chains of plasma channels, such as beam quality preservation, hosing and beam-break-up instabilities.

The exascale challenge problem is the modelling of a chain of tens of plasma acceleration stages. Realizing such an ambitious target is essential for the longer-range goal of designing a single- or multi-TeV electron-positron high-energy collider based on plasma acceleration technology [43]. The WarpX application uses AMReX for mesh refinement and employs PIC methodology to solve the dynamic Maxwell equations to model the accelerator system. The objective is the modelling of multi-TeV high-energy physics colliders based on tens to hundreds of plasma-based accelerator stages. This will be achieved by modelling of an increasing number of consecutive stages to reach higher final energy, and to increase the precision (and hence confidence) of the numerical results by performing simulations at higher resolutions, in a reasonable clock time.

## 6. Earth and space science applications

The Earth and Space Science Application area spans fundamental scientific questions from the origin of the universe and chemical elements to planetary processes and interactions affecting life and longevity. This area addresses phenomena where controlled and fine resolution data collection is extremely difficult or infeasible, and, in many cases, fundamental simulations are the best source of data to confirm scientific theories and predict critical phenomena.

### (a) Stellar explosions

Astronomical observations have confirmed that the production of heavy elements occurred early in galactic history. Yet many details remain outside the purview of direct observation. Exascale computing, through the *ExaStar* application and its Clash code suite (https://sites.google.com/lbl.gov/exastar), will help address fundamental questions in astrophysics, including understanding the origin of elements. ExaStar will employ exascale computing to help coordinate experimental observations to gain a fuller understanding of where and how heavy elements are born.

ExaStar's new code suite (Clash) is a componentized multi-physics AMR-based toolkit designed for the high-fidelity simulation of coupled hydrodynamics, radiation transport, thermonuclear kinetics and nuclear microphysics for analysis of stellar explosion and other related phenomena (e.g. neutron star mergers). Clash will reach exascale efficiency by building upon current many-core efficient local physics packages integrated into a task-based asynchronous execution framework based on AMR technology. The fundamental goal in the development of Clash is to understand the production of the chemical elements in these explosions, particularly those heavier than iron. While astronomical observations reveal the production of the heaviest nuclei began early in galactic history, it is not known how and where these elements were formed. To address this topic via laboratory measurements, a series of Nuclear Science Long-Range Plans [44] have supported construction of radioactive ion beam facilities, culminating in the Facility for Rare Isotope Beams (FRIB). While FRIB is designed to acquire extensive data on the nuclei relevant for astrophysical nucleosynthesis, its end science goal cannot be met unless those experimental data are integrated into high-fidelity simulations of stellar explosions—supernovae and neutron star mergers—that define the conditions under which such heavy element production most likely takes place. Through a better understanding of the sites where the heaviest elements are made, ExaStar will help focus FRIB experimental efforts on those reactions of greatest influence.

The ExaStar exascale challenge problem is a three-dimensional simulation of the first 2 s of evolution after iron core bounce of a core-collapse supernova. Candidate progenitor star models are the solar metallicity 12 solar mass progenitor of Sukhbold *et al*. [45], because it represents, in some sense, the ‘centre’ of the distribution of massive stars that produce core-collapse supernovae (CCSNe); or the binary merger model of Menon & Heger [46], as it is likely to closely mimic the progenitor system of SN 1987a, the only CCSNe to date with the presence of multi-messenger signals. The ExaStar computational domain will extend from the centre of the star out to fully enclose the helium shell of the evolved star. The precise location of this radius is progenitor-dependent, but is generally more than 10 000 km. The maximum spatial resolution (enabled with AMR) will be at least 1 km at the surface of the proto-neutron star (i.e. in the inner 100 km or so of the event). At least 20 energy groups must be used to resolve the spectra of neutrinos of all flavours (electron, mu, tau and their anti-particles) from 0 to 300 MeV. An approximation to general relativistic gravity using at least 12 moments in a multipole approach will be used, with a more realistic treatment (e.g. dynamical general relativity) possible. A set of tabulated neutrino–matter interaction rates that include emission, absorption, scattering and pair production from various nuclear and nucleonic processes will be used. This table will be coupled to a set of tabulated quantities derived from a high-density equation of state for pressures, entropies and all other required thermodynamic values as required by, e.g., the hydrodynamics.

### (b) Computing the sky at extreme scales

Modern cosmological observations carried out with large-scale sky surveys are unique probes of fundamental physics. They have led to a remarkably successful model for the dynamics of the universe as well as a number of breakthrough discoveries leading to multiple Nobel prizes. Three key ingredients—dark energy, dark matter and inflation—are signposts to further breakthroughs, as all reach beyond the known boundaries of the Standard Model of particle physics. Sophisticated, large-scale simulations of cosmic structure formation are essential to this scientific enterprise. They not only shed light on some of the deepest puzzles in all of physical science but also rank among the very largest and most scientifically rich simulations run on supercomputers today. The *ExaSky* application is extending existing cosmological simulation codes to efficiently execute on exascale platforms for this simulation challenge.

A new generation of sky surveys will provide key insights into questions raised by the current paradigm as well as provide new classes of measurements, such as of neutrino masses. They may lead to exciting new discoveries, including that of primordial gravitational waves and modifications of general relativity. Existing HPC systems do not have the performance and the memory needed to run the next-generation simulations that are required to meet the challenge posed by near-future surveys. ExaSky extends the HACC [47] and Nyx [48] cosmological simulation codes to efficiently use exascale resources as they become available. The Eulerian AMR code Nyx complements the Lagrangian nature of HACC; the two codes are being used to develop a joint program for verification of gravitational evolution, gas dynamics and astrophysical subgrid models in cosmological simulations at a very high dynamic range.

Statistical and systematic error requirements on a large number of cosmological summary statistics exist to establish accuracy baselines. These statistics include the density fluctuation power spectrum, the halo mass function (dark matter forms localized clumps, within which galaxies forms, these clumps are called ‘halos’), the halo bias (the distribution of halos relative to the distribution of the overall mass) as a function of mass, the weak gravitational lensing shear power spectrum, and kinematic and thermal Sunyaev–Zel'dovich effects for galaxy clusters (halos hosting hot gas with temperatures in the millions of degrees). There are also a number of cross-correlations such as the density-halo cross power, and cosmic microwave background cross-correlation with large-scale structure. The accuracy requirements are typically scale-dependent, large spatial scales being subject to finite-size effects and small scales being subject to a number of more significant problems such as particle shot noise and code evolution errors (including subgrid modelling biases). Strict accuracy benchmarks have already been set by the observational requirements for surveys such as CMB-Stage 4, Dark Energy Spectroscopic Instrument (DESI) and the Large Synoptic Survey Telescope (LSST), which typically are sub-per cent (statistical) over the range of well-observed scales. Systematic errors need to be characterized, and controlled where possible, to the per cent level or better. ExaSky exascale challenge problems must be carried out with a new set of subgrid models for gas cooling, UV heating, star formation and supernova and active galactic nucleus feedback.

Required simulation sizes are set by the scales of the cosmological surveys. Exascale challenge problem simulations must cover boxes of linear size up to the several Gpc scale, with galaxy formation-related physics modelled down to roughly 0.1 kpc (a dynamic range of one part in 10 million, improving the current state of the art by an order of magnitude). Multiple-size boxes will be run to cover the range of scales that need to be robustly predicted. The mass resolution of the simulations (in the smaller boxes) will go down to roughly a million solar masses for the baryon tracer particles and about five times this value for the dark matter particles. The final dynamic range achieved depends on the total memory available on the first-generation exascale systems.

ExaSky simulation suites fall into three categories: (1) large-volume, high mass and force resolution gravity-only simulations; (2) large-volume, high mass and force resolution hydrodynamic simulations including detailed subgrid modelling; and (3) small-volume, very high mass and medium/high force resolution hydrodynamic simulations including subgrid modelling. The first set of simulations is targeted at DESI observations of luminous red galaxies, emission line galaxies, quasars and for end-to-end simulations for LSST. The second (main) set of simulations will include hydrodynamics and detailed subgrid modelling with the resolution and physics reach improving over time as more powerful HPC systems arrive. The main probes targeted with these simulations are strong and weak lensing shear measurements, galaxy clustering, clusters of galaxies and various cross-correlations. The third set consists of smaller volume, hydrodynamic simulations for convergence testing and verification and for developing and testing a new generation of subgrid models based on results from high-resolution, small effective volume, galaxy formation studies.

### (c) Regional-scale earthquake hazard and risk assessment

Large earthquakes present a significant risk around the world and are also a concern for DOE ranging from the safety of DOE's own inventory of one-of-a-kind mission-critical facilities to all major US energy systems. In general, addressing earthquake risk, both from the standpoint of safety, damage and economic impact, is a major societal challenge for virtually every element of the built environment, including transportation, health, data, commerce and all urban infrastructure. The *EQSIM* application is tapping HPC developments, data collection and data exploitation to advance earthquake hazard and risk assessments. EQSIM application codes are removing the reliance on simplifying idealizations, approximations and sparse empirical data by focusing on resolving the fundamental physics uncertainties in earthquake processes. Through EQSIM, regional-scale ground motion simulations are becoming computationally feasible, and simulation models that connect the domains of seismology, geotechnical and structural engineering are becoming within grasp.

The EQSIM application is focused on creating an unprecedented computational toolset and workflow for earthquake hazard and risk assessment [49]. Starting with a set of the existing codes, SW4 (a fourth order, three-dimensional seismic wave propagation model), NEVADA (a nonlinear, finite displacement program for building earthquake response) and ESSI (nonlinear finite-element program for coupled soil-structure interaction), EQSIM is building an end-to-end capability to simulate from the fault rupture to surface ground motions (earthquake hazard) and ultimately to infrastructure response (earthquake risk). The ultimate goal of the EQSIM development is to remove computational limitations as a barrier to scientific exploration and understanding of earthquake phenomenology, as well as to practical earthquake hazard and risk assessments.

Traditional earthquake hazard and risk assessments for critical facilities have relied on empirically based approaches that use historical earthquake ground motions from many different locations to estimate future earthquake ground motions at a specific site of interest. Given the fact that ground motions for a particular site are strongly influenced by the physics of the specific earthquake processes including the fault rupture mechanics, seismic wave propagation through a heterogeneous medium and site response at the location of a particular facility, earthquake ground motions are very complex with significant spatial variation in both frequency content and amplitude. The homogenization of many disparate records in traditional empirically based ground motion estimates cannot fully capture the complex site-specificity of ground motion. Over the past decade, interest in using advanced simulations to characterize earthquake ground motions and infrastructure response has accelerated significantly. However, the extreme computational demands required to execute hazard and risk simulations at regional scale have been prohibitive. A fundamental objective is to advance regional-scale ground motion simulation capabilities from the historical computationally limited frequency range of up to 2 Hz, to the frequency range of interest for a breadth of engineered infrastructure of up to 10 Hz. A second fundamental objective is to implement an HPC framework and workflow that directly couples earthquake hazard and risk assessments through an end-to-end simulation framework that extends from earthquake rupture to structural response, thereby capturing the complexities of interaction between incident seismic waves and infrastructure systems.

To achieve the overall goals, regional-scale forward ground motion simulations must be executed at unprecedented frequency resolution with much larger, much faster models. Achieving fast earthquake simulation times is essential to allowing the parametric variations necessary to span critical problem parameters (e.g. multiple fault rupture scenarios). Second, as the ability to compute at higher frequencies progresses, better characterization of subsurface geologic structure at finer and finer scales is needed, thus a companion schema for representing fine-scale geologic heterogeneities in massive computational models must be developed. For the purpose of evaluating regional-scale simulations and assessing progress, a representative large regional-scale model of the US San Francisco Bay Area is being targeted that includes all necessary geophysics modelling features (three-dimensional geology, earth surface topography, material attenuation, nonreflecting boundaries, fault rupture models) [50]. For a 10 Hz simulation, the computational domain requires 200–300B grid points as a basis for testing and evaluating advanced physics algorithms and implementations.

### (d) Subsurface wellbores and fractures

Understanding and predicting reservoir-scale behaviour as affected by the long-term integrity of the hundreds of deep wells that penetrate the subsurface is important for safe and appropriate resource utilization. The performance of a wellbore hinges on the behaviour of very thin interface features controlling the leakage of fluids along the well casing–cement boundary. Similarly, leakage of buoyant fluids (e.g. CO_2_) through caprocks may be controlled by micrometre-scale asperities in fracture networks that are themselves subject to geomechanical and geochemical modification. At the reservoir or field scale (approx. 1–10 km), multiphase flow and reactions in fractured porous media are typically modelled using continuum models that make use of averaged quantities and bulk parameters that do not fully account for heterogeneity at different spatial and temporal scales owing to coupled thermal, hydrological, chemical and mechanical processes. A more rigorous treatment resolves the pore-scale (0.1–10 µm) physical and geochemical heterogeneities in wellbores and fractures to improve predict of the evolution of these features when subjected to geomechanical and geochemical stressors, since these features ultimately control the reservoir-scale permeability and reactivity. The *Subsurface* application is using exascale to integrate these complex multi-physics processes occurring at multiple scales (micrometre to kilometre) into a high-resolution reservoir simulator.

There are a wide range of processes that take place in the subsurface that involve the evolution of fractures, including both opening and closing due to some combination of mechanical and chemical stresses. The *Subsurface* application is focused on the failure of a wellbore for CO_2_ sequestration in saline reservoirs, with consideration of a wellbore segment of up to 100 m, and time periods up to 1 year. Wells are considered to be high-risk pathways for fluid leakage from geologic CO_2_ storage reservoirs, because breaches in this engineered system have the potential to connect the reservoir to groundwater resources and the atmosphere. A concern in the geologic carbon storage community is wellbore stability—because acidic fluids in the CO_2_ storage reservoir, alkaline cement meant to isolate the reservoir fluids from the overlying strata, and steel casings in wells are all inherently reactive systems. This is of particular concern for storage of CO_2_ in depleted oil and gas reservoirs with numerous legacy wells engineered to variable standards.

In contrast to the conventional treatment of wellbore failure that is currently modelled at large scales on the order of 100–1000 m and 10 years, accurate prediction of fracture evolution depends on microscale resolution of fracture asperities (pillars) controlling permeability and chemical reactivity. Microscale resolution is also needed to accurately predict fracture permeability, since real rough fractures are typically held open by asperities (pillars) of this scale. Chemical corrosion (dissolution) or mechanical corrosion (pressure solution) of these asperities occurs at the same micrometre scale. The localized subdomain needed to resolve reactive transport processes at microscale resolution during fracture propagation is a domain size up to 10 cm (in the length of the wellbore) × 1 cm (along an azimuth in the cement annulus) × 1 mm (in the radial direction) with 1 µm grid resolution. This is the minimum domain needed to capture coupled reactive transport and mechanics effects in a fracture (e.g. pillar collapse).

The Subsurface application addresses this exascale challenge problem by coupling two mature code bases: (i) Chombo-Crunch [51–53], which models Navier–Stokes and Darcy flow coupled to multicomponent geochemical reaction networks, and (ii) GEOSX [54], which models geomechanical deformation and fracture + Darcy flow at a variety of scales. The focus is on the evolution of a single fracture in wellbore cement, beginning with diffusion-controlled reaction and weakening of the cement that leads to fracturing. The propagation of the fracture resulting from further chemical reaction and fluid pressure-driven deformation is simulated with 1 µm resolution within the fracture and is coupled to a coarser resolution (10 µm) representation of the porous cement adjacent to the evolving fracture. This model will require greater than 1T grid cells with 16T degrees of freedom to account for the appropriate hydraulic, mechanical and chemical phenomena.

### (e) Cloud-resolving earth system model

The goal of the *E3SM-MMF* application is to develop a cloud-resolving earth system model with throughput necessary for multi-decade, coupled high-resolution climate simulations. This next-generation model has the potential to substantially reduce major systematic errors in precipitation found in current models because of its more realistic and explicit treatment of convective storms. The goal is to improve the ability to assess regional impacts of climate change on the water cycle, and its commensurate impacts on agriculture and energy production. The impact of climate change on the global and regional water cycle is one of the highest priorities and most difficult challenges in climate change prediction.

Current earth system models possess limited ability to model the complex interactions between the large scale, mostly two-dimensional baroclinic atmospheric motions and the smaller scale three-dimensional convective motions found in clouds and individual storms. These motions and their interactions, to first order, determine the spatial distributions and characteristics of regional precipitation. Complexities include the microscale chemistry and physics of cloud formation and the impacts of anthropogenic climate change on cloud formation. Properly resolving the key processes involved in cloud formation requires resolution (grid spacing) of the order of 1 km in the atmosphere. It is possible to run such resolution on today's petascale computing systems, but only at great expense and for very short times (several simulated days). Running conventional climate models at this resolution, for 100-year simulations, requires a 5000 × increase in computing resources.

At exascale, the ESSM-MMF application is adopting a multiscale modelling framework (MMF) methodology to cloud-resolving modelling, often referred to as super-parametrization, which offers significant opportunities for unprecedented model skill improvement that has yet to be fully explored due to limited computing resources. The approach implements a cloud-resolving convective parametrization (super-parametrization) into the DOE Energy Exascale Earth System Model (E3SM, http://e3sm.org) using the MMF and explores its full potential to scientifically and computationally advance climate simulation and prediction. The super-parametrization is designed to make full use of GPU accelerated systems and will also involve refactoring and porting other key components of the E3SM model for GPU systems.

The subsequent exascale challenge problem has several aspects: achieving cloud-resolving resolution in the atmosphere super-parametrization, which is defined as at least 1 km grid spacing in both horizontal and vertical directions; achieving weather resolving resolution in the global atmosphere model, which is defined as 50–25 km average grid spacing in the horizontal directions with approximately 1 km grid spacing in the vertical directions (the resolution of today's global operational forecast models); and achieving an eddy-resolving ocean/ice model, which is defined as a minimum 18 km resolution in equatorial regions, decreasing to 6 km in polar regions. The final aspect is achieving model throughput necessary to perform the simulation campaign in the course of one calendar year on the exascale Frontier system.

## 7. Data analytics and optimization applications

The data analytics and optimization application area includes applications whose predictive capability is in part based on modern data analysis and machine learning techniques rather than strictly on approximate solutions to equations that reflect fundamental physical principles or reduced semiempirical models. These applications include a broad range of domain areas and techniques, some of which are only recently coming into maturity in the context of high-end simulation.

### (a) Stochastic power grid dynamics

Maintaining the integrity of power grids under adverse conditions imposed by natural or man-made causes is critical for national and economic security. When power grids are subject to localized stresses, load imbalances can occur between electricity supply and demand at the system level. The *ExaSGD* application is developing models and algorithms to help optimize the grid's response (ideally in real time) against potential disruption events. ExaSGD will harness the power of exascale computing to help power grid planners and operators maintain power grid integrity under emergency conditions.

Power grids operate by maintaining balance between electricity supply and demand. Electricity is produced at generators via fossil and nuclear fuels, hydro resources, renewables or other sources, and is transmitted through a bulk power system in the USA at a 60 Hz frequency. Attacks (via physical or cyber means) and hazards on the grid create an imbalance between supply and demand, which can result in drops in frequency, which can result in large-scale blackouts and/or permanently damage very large and expensive components. Great care must, therefore, be taken to operate the power grid with very high reliability within narrow operating frequency ranges.

Recovering from generation-load imbalance can be achieved by shedding load (deliberately allowing some load to go unserved creating a partial blackout) to preserve the functionality of the remainder of the power grid. But the behaviour of the power grid can be influenced at many points within the system because of the increasing prevalence of cyber-enabled control and sensing, renewables (e.g. transient wind or solar power), plug-in storage devices (e.g. electric vehicles), smart metres to control load at a fine granularity (e.g. throttling home appliances at times of peak demand), and other sensored elements controlled remotely. Current practice focuses primarily on a conventional load shedding approach, where portions of the system are disconnected to remove the load they impose. The resulted loss of load is not desirable outcomes, especially when the load is shed at a widespread scale because the grid is heavily stressed. The goal is to minimize such loss of load while still maintaining the resilience of the power grid. The ExaSGD application is exploring more efficient strategies for dynamically achieving balance using a more complete spectrum of grid elements besides load shedding. A simulation capability for discovering more optimal configurations to recover from generation-load imbalance is being developed to improve readiness to recover from a variety of hazards to the power grid.

The ExaSGD exascale challenge problem is to optimize power grid response in a near-term timeframe (e.g. 30 minutes) to a variety of underfrequency hazards via physical and control threat scenarios using comprehensive modelling that includes generation, transmission, load and cyber/control elements. This capability enables efficient analysation of numerous sampled hazards to quickly discover strategies to reestablish balance with minimal impact to loads served. ExaSGD will compare the frequency recovery performance of a complex grid plus control system in the presence and the absence of smart devices, stored energy reserves, renewables and demand response technologies. This will involve multiple simulations of the distribution of severity of frequency response to grid hazard and the resulting effects. Estimating these distributions involves the solution to a large number of optimal power flow calculations that consider different underfrequency scenarios. Each optimal power flow calculation requires the solution to a large-scale nonlinear optimization problem. This challenge problem will also consider the integrated execution of these optimization problems to warm-start subsequent power flow calculations across scenarios. The ultimate outcome is to maintain the generation-load balance without a large-scale loss of load and make the power grid resilient to cyber and man-made disruptions.

### (b) Deep learning enabled precision medicine for cancer

The DOE has entered into a partnership with the National Cancer Institute (NCI) of the National Institutes of Health (NIH). This partnership has identified three key challenges that the combined resources of DOE and NCI can accelerate. The first challenge (called the ‘drug response problem’) is to develop predictive models for drug response that can be used to optimize pre-clinical drug screening and drive precision medicine-based treatments for cancer patients. The second challenge (called the ‘RAS pathway problem’) is to understand the molecular basis of key protein interactions in the RAS/RAF pathway that is present in 30% of cancers. The third challenge (called the ‘treatment strategy problem’) is to automate the analysis and extraction of information from millions of cancer patient records to determine optimal cancer treatment strategies across a range of patient lifestyles, environmental exposures, cancer types and healthcare systems. While each of these three challenges are at different scales and have specific scientific teams collaborating on the data acquisition, data analysis, model formulation and scientific runs of simulations, they also share several common threads. The *CANDLE* application project focuses on supporting scalable deep learning aspects of the three challenge problems and, in particular, is constructing a scalable deep learning environment for exascale systems called CANDLE (CANcer Distributed Learning Environment). Further information is at https://candle.cels.anl.gov and https://github.com/ECP-CANDLE/Benchmarks.

CANDLE has three specific strategies to address these three challenges. For the drug response problem, supervised machine learning methods are used to capture the complex, nonlinear relationships between the properties of drugs and the properties of the tumours to predict response to treatment (and therefore develop a model that can provide treatment recommendations for a given tumour). For the RAS pathway problem, multi-scale MD runs are guided through a large-scale state-space search using unsupervised learning to determine the scope and scale of the next series of simulations based on the history of previous simulations. For the treatment strategy problem, semi-supervised machine learning is used to automatically read and encode millions of clinical reports into a form that can be computed upon. Each problem requires a different approach to the embedded learning problem, all of which are supported with the same scalable deep learning environment in CANDLE.

The CANDLE software suite broadly consists of three components: the collection of deep neural networks that capture and represent the three problems, a python library that extends the popular TensorFlow and PyTorch environments for exascale level computing and the runtime supervisor component that orchestrates work distribution across an HPC system. The CANDLE python library provides a series of functions that streamline the process of implementing CANDLE-compliant code. The functionality includes support for large-scale search of hyperparameters, that enables automatic searching for better model performance, support for semi-automated uncertainty quantification, so that model predictions can have associated confidence values, support for neural architecture search methods aimed at expanding strategies for improved model discovery and methods for large-scale ensembles, complex workflows, and model and data parallelism. CANDLE is also working on additional library extensions that will support transfer learning, Bayesian neural networks and active learning. Each of these capabilities are being integrated into a single environment that has been ported to multiple architectures in the DOE computing complex. CANDLE is also deployed on the computing clusters at the NIH and the NCI.

The challenge for exascale is manifested in the need to rapidly train large numbers of related models. A need inherent to each pilot application is producing optimized models that cover the space of specific predictions (individualized in the precision medicine sense). Take, for example, training a model that is specific to a certain drug and individual cancer. Starting with 1000 different cancer cell lines and 1000 different drugs, a leave-one-out strategy to create a high-resolution model for all drug by cancers requires approximately one million models. Yet, these models are similar enough that using an incremental learning strategy where weights are shared during training in a way that avoids information leakage can significantly reduce the time needed to train a large set of models.

### (c) Microbiome analysis

Microbial species live in, on and around plants, animals, soil, oceans and the atmosphere and have a critical role in the health of individuals and the environment. They can affect agricultural production, perform environmental remediation and be used to manufacture fuels, medicines and other products. Yet microbial species are not well understood and many cannot be studied in isolation, but only as part of a naturally occurring community. Genome sequencing on DNA extracted from microbiomes is used to study the diversity, integration and dynamics of these organisms. Owing to the size and complexity of these datasets, genome assembly and comparative analysis are some of the most computationally demanding aspects of bioinformatics. Furthermore, as genome sequencing technology continues to improve, metagenomic data are both larger and more abundance, so the computational cost will only grow. The *ExaBiome* application project (http://exabiome.org) is developing scalable data assembly and analysis tools to address current needs and, through the use of exascale computing power, provide solutions for anticipated increases in biological data.

Metagenomics is the application of high-throughput genome sequencing technologies to DNA extracted from microbiomes in a naturally occurring community which may have hundreds of individual microbial species. Since the introduction of metagenomics over a decade ago, it has become an essential tool in understanding the make-up and function of the microbiome. ExaBiome aims to provide scalable tools for three core computational problems in metagenomics: (i) metagenome assembly, which takes raw sequence data and produces long genome sequences for each species; (ii) protein clustering, which finds families of closely related proteins; and (iii) signature-based approaches to enable scalable and efficient comparative metagenome analysis, which may show variability of an environmental community over time or the impact of other environmental factors such as temperature or moisture.

The ExaBiome team has developed a scalable metagenome assembler, MetaHipMer, which scales well on thousands of compute nodes on today's petascale-class architectures and has already assembled large environmental datasets that had not been possible with previous tools. Work continues on further scalability improvements across nodes and new node-level optimizations to take advantage of fine-grained on-node parallelism and memory structures, including GPUs. MetaHipMer exhibits competitive quality with other assemblers, and the ExaBiome team continues to add innovations and parameters to control various aspects of how the data are analysed, driven by the experience of science teams. MetaHipMer is designed for short read (Illumina) data, but a second assembler for long reads is also under development and shows even higher computational intensity, which may be a good fit for exascale systems. A second ExaBiome code, HipMCL, provides scalable protein clustering. HipMCL runs on thousands of nodes and has already been used to provide insight on the structure of protein families across hundreds of millions of proteins, a dataset that was previously intractable. These codes and comparative analysis tools use some common computational patterns, including dynamic programming for string alignment (either DNA or proteins) with minimal edits, counting and analysis of fixed-length strings (k-mers), and a variety of graph and sparse matrix methods. Components of these codes are also being developed for comparative analysis tools.

The ExaBiome exascale challenge problem focuses on metagenome assembly, but that capability will enable exascale feasibility for other bioinformatics problems in ExaBiome and more broadly. The challenge problem is to demonstrate a high-quality assembly or set of assemblies on at least 50TB of environmental data (reads) that runs across a full exascale machine. The intent is to use a scientifically interesting environmental sample that may include multiple temporal or spatial samples and to be performed as a single assembly using complete sequence data. By contrast, current state-of-the-art assembly pipelines are forced to use subsampling when datasets get large, which limits the ability to assemble rare, low-coverage species and with repeated region of their genomes. Furthermore, assembling data across time and spatial scales together will not only enhance the assembly quality, but could reveal functions that otherwise would remain hidden. Addressing this challenge problem will demonstrate a first-in-class science capability using the power of exascale computing combined with novel graph algorithms. There are many potential beneficial science impacts, for example enhancing understanding of microbial functions that can aid in environmental remediation, food production and medical research.

### (d) Data analytics for free-electron lasers

The Linac Coherent Light Source (LCLS) facility at the Stanford Linear Accelerator Center (SLAC) uses X-ray diffraction to image individual atoms and molecules in order to observe fundamental processes in physics, chemistry and biology. Near real-time interpretation of molecular structure revealed by X-ray diffraction will require computational intensities of unprecedented scales coupled to a data path of unprecedented bandwidth. Detector data rates at light sources are advancing exponentially: The LCLS will increase its data throughput by three orders of magnitude by 2025 after the LCLS-II-HE upgrade is complete. The objective of the *ExaFEL* application (https://lcls.slac.stanford.edu/exafel) is to effectively use exascale computing to reduce the time (from weeks to minutes) to reconstruct molecular structures from X-ray diffraction data.

Users of the LCLS require an integrated combination of data processing and scientific interpretation, where both aspects demand intensive computational analysis. The ultrafast X-ray pulses are used like flashes from a high-speed strobe light that produce stop-action movies of atoms and molecules. The analysis must be carried out quickly to allow users to iterate their experiments and extract the most value from scarce beam time. Enabling new photon science from the LCLS will require near real-time analysis (approx. 10 min) of data bursts, requiring commensurate bursts of exascale-class computational intensities.

The high repetition rate and ultra-high brightness of the LCLS make it possible to determine the structure of individual molecules, mapping out their natural variation in conformation and flexibility. Structural dynamics and heterogeneities, such as changes in size and shape of nanoparticles, or conformational flexibility in macromolecules, are at the basis of understanding, predicting and eventually engineering functional properties in biology, material and energy sciences. The ability to image these structural dynamics and heterogeneities using non-crystalline based diffractive imaging, including single-particle imaging (SPI) and fluctuation X-ray scattering, has been one of the driving forces of the development of X-ray free-electron lasers. However, efficient processing of the data, classification of diffraction patterns into conformational states and the subsequent reconstruction of a series of three-dimensional electron densities, which allow one to visualize how the structure is changing, are vital computational challenges in diffractive imaging.

The ExaFEL challenge problem is the creation of an automated analysis pipeline for imaging of single particles via diffractive imaging. This requires reconstruction of three-dimensional molecular structure from two-dimensional diffraction images using the new Multi-Tiered Iterative Phasing (M-TIP) algorithm. In SPI, diffraction images are collected from individual particles, and are used to determine molecular (or atomic) structure, even from multiple conformational states (or non-identical particles) under operating conditions. Determining structures from SPI experiments is challenging, since orientations and states of imaged particles are unknown, and images can be highly contaminated with noise. Furthermore, the number of useful images is often limited by achievable single-particle hit rates. The M-TIP algorithm introduces an iterative projection framework to simultaneously determine orientations, states and molecular structure from limited single-particle data by leveraging structural constraints throughout the reconstruction, offering a potential pathway to increasing the amount of information that can be extracted from single-particle diffraction.

Rapid feedback is crucial for tuning sample concentrations to achieve a sufficient single-particle hit rate, ensuring that adequate data are collected and steering the experiment. The availability of exascale computing resources and an HPC workflow that can handle incremental bursts of data in the analyses will allow researchers to perform data analysis on the fly, providing immediate feedback on the quality of the experimental data, while determining the three-dimensional structure of the sample at the same time.

## 8. National security applications

The National Security Applications projects are developing next-generation multi-physics simulations tools that address emerging HPC challenges of massive heterogeneous parallelism in support of the mission of the stockpile stewardship program (SSP) (https://www.energy.gov/nnsa/missions/maintaining-stockpile). The projects are part of the Advanced Technology Development and Mitigation (ATDM) program element of the DOE National Nuclear Security Administration (NNSA) Advanced Simulation and Computing (ASC) Program and are implemented at each of the three DOE NNSA Laboratories. Each project has a specific challenge problem target, namely a currently intractable three-dimensional problem of interest. For Los Alamos National Laboratory (LANL) and Lawrence Livermore National Laboratory (LLNL), one of the demonstration applications includes multi-physics simulation of high-energy density physics for inertial confinement fusion. For Sandia National Laboratories (SNL), the demonstration applications require solving the multi-physics phenomena in re-entry aerodynamics and electromagnetic plasma physics. All four applications must exhibit efficient use of one or more of the architecture of the ASC Advanced Technology Systems located at the NNSA laboratories as well as portability and high confidence in physics fidelity. Scalability has been a design priority from the beginning, and the ability of the new codes to scale to some significant fraction of future machines is an objective. High confidence in physics fidelity has also been an objective from the beginning, with development of novel multi-scale and high-order algorithms and integrated verification and validation.

The outcomes and products of this activity will be integrated into the next generation of integrated and high-performance ASC codes on advanced (next decade) architectures in support of the vast NNSA mission scope. The LANL approach is to concurrently develop a flexible framework, code infrastructure and physics components with multi-scale algorithms [55–57]. LLNL is developing new high-order algorithms to minimize data motion relative to computation, which are then incorporated into production codes built on modular, interoperable software layers [58]. The SNL approach is built upon agile components as part of a comprehensive toolkit, which includes a data model, an abstraction layer, discretization techniques and high-quality solvers implemented with performance portable abstractions [59–62]. The ECP NNSA Applications work closely with ECP software technologies team at each laboratory. Together, the three NNSA laboratories aim to deliver applications that can address currently infeasible three-dimensional problems of interest. These different approaches are complementary, providing both peer review and risk mitigation.

## 9. Conclusion

The ECP is posed to deliver science-based computational and data science applications that effectively exploit exascale HPC technologies to provide breakthrough modelling and simulation solutions, yielding high-confidence insights and answers to the nation's most critical problems and challenges. Given the stated challenge problem goals for ECP applications, their collective development is nevertheless difficult, challenging and risky. But with high risk often comes high rewards, as is the case if ECP applications can adequately address their challenge problems (hence ‘skin in the game’).

ECP applications, including both their technologies and solutions, will have a profound impact on the strength of the nation and the quality of life for all citizens. Examples of their expected outcomes and impact, which will be far-reaching for decades to come, include:

— Predictive microstructural evolution of novel chemicals and materials for energy applications.
— Robust and selective design of catalysts an order of magnitude more efficient at temperatures hundreds of degrees lower.
— Accelerate the widespread adoption of additive manufacturing by enabling the routine fabrication of qualifiable metal alloy parts.
— Design next-generation quantum materials from first principles with predictive accuracy.
— Predict properties of light nuclei with less than 1% uncertainty from first principles.
— Harden wind plant design and layout against energy loss susceptibility, allowing higher penetration of wind energy.
— Demonstrate commercial-scale transformation energy technologies that curb fossil fuel plant CO_2_ emission by 2030.
— Accelerate the design and commercialization of small and micronuclear reactors.
— Provide a ‘whole device’ modelling capability for magnetically confined fusion plasmas required to design and operate ITER and future fusion reactors.
— Address fundamental science questions such as the origin of elements in the universe, the behaviour of matter at extreme densities, the source of gravity waves; and demystify key unknowns in the dynamics of the universe (dark matter, dark energy and inflation).
— Reduce the current major uncertainties in earthquake hazard and risk assessments to ensure the safest and most cost-effective seismic designs.
— Reliably guide safe long-term consequential decisions about carbon storage and sequestration.
— Forecast, with confidence, water resource availability, food supply changes and severe weather probabilities in our complex earth system environment.
— Optimize power grid planning and secure operation with very high reliability within narrow operating voltage and frequency ranges.
— Develop treatment strategies and pre-clinical cancer drug response models and mechanisms for RAS/RAF-driven cancers.
— Discover, through metagenomics analysis, knowledge useful for environment remediation and the manufacture of novel chemicals and medicines.
— Dramatically cut the cost and size of advanced particle accelerators for various applications impacting our lives, from sterilizing food of toxic waste, implanting ions in semiconductors, developing new drugs or treating cancer.

## Data accessibility

This article has no additional data.

## Disclaimer

This manuscript has been authored by UT-Battelle, LLC under Contract No. DE-AC05-00OR22725 with the US Department of Energy. The United States Government retains and the publisher, by accepting the article for publication, acknowledges that the United States Government retains a non-exclusive, paid-up, irrevocable, worldwide license to publish or reproduce the published form of this manuscript, or allow others to do so, for United States Government purposes. The Department of Energy will provide public access to these results of federally sponsored research in accordance with the DOE Public Access Plan (http://energy.gov/downloads/doe-public-access-plan). This work was also authored in part by the National Renewable Energy Laboratory, operated by Alliance for Sustainable Energy, LLC, for the U.S. Department of Energy (DOE) under Contract No. DE-AC36-08GO28308.
